# Population‐based approaches for monitoring the nurturing care environment for early childhood development: A scoping review

**DOI:** 10.1111/mcn.13276

**Published:** 2021-11-04

**Authors:** Jéssica Pedroso, Stefanie Eugênia dos Anjos Coelho Kubo, Priscila Olin Silva, Gabriel Ferreira de Castro, Juliana Lopes Pimentel, Rafael Pérez‐Escamilla, Muriel Bauermann Gubert, Gabriela Buccini

**Affiliations:** ^1^ Departamento de Nutrição Universidade de Brasilia Brasilia Brazil; ^2^ Department of Social and Behavioral Sciences Yale School of Public Health New Haven Connecticut USA; ^3^ Department of Environmental and Occupational Health University of Nevada Las Vegas Las Vegas Nevada USA

**Keywords:** child, child development, environment, index, infant, nurturing care, public health surveillance

## Abstract

Selecting indicators to monitor nurturing care (NC) environments that support decision‐making and guide the implementation of integrated early childhood development (ECD) programmes has become a priority globally. Several population‐based approaches have been attempted to create a set of indicators or a composite index methodology to measure the NC environment using existing secondary data. However, they have not been systematized. Our scoping review aimed to analyse the population‐based approaches for monitoring the domains of the NC (e.g. good health, adequate nutrition, responsive caregiving, security and safety, and opportunities for early learning). ECD experts, peer‐reviewed, and grey literature were systematically searched with no year or language restrictions. Data extraction used a standard predefined protocol. Thirty‐two population‐based approaches were identified. Most approaches were composed of a set of indicators (53.1%, *n* = 17) versus composite indexes (46.9%, *n* = 15) and had the country as their unit of analysis (68.8%, *n* = 22). Twenty‐seven approaches were applied in middle‐income countries (84.4%) and thirteen in low‐income countries (40.6%). Four approaches were guided by the NC framework (12.5%), and 56.3% (*n* = 18) did not include any indicator representing responsive caregiving. NC indicators (*n* = 867) were sorted into 100 groups of indicators. Twenty of the 32 approaches had some kind of methodological validation (62.5%). We identified six methodological challenges to build a population‐based approach. Standardized methods for selecting and validating indicators, and coordinated efforts to share findings/data with stakeholders should be prioritized. Given the great variability in methods and indicators used to measure NC environments, valid approaches should be flexible to work well across different contexts.

**Key messages**

- Given the great variability in methods used to measure the nurturing care (NC) environment, valid approaches based on reliable indicators should be prioritized.
- NC monitoring approaches need to be flexible to fit across different contexts and governance levels, from national to state and local levels.
- Most monitoring approaches lacked a robust equity approach.
- Disaggregated data is one of the most critical gaps for measuring the NC.
- User‐friendly NC monitoring systems are needed by decision makers. Future research should assess the usefulness of such population‐based approaches to improve NC environments for ECD.

## 1 INTRODUCTION

Early childhood is a phase characterized by intense neurogenesis and brain plasticity in response to nurturing care (NC) (Clark et al., 2020; World Health Organization et al., 2018). The NC framework calls for safe, secure, and stimulating environments where children have opportunities to learn and interact with caregivers that are emotionally supportive, sensitive, and responsive to their developmental and physiological needs (Black et al., 2017; Pérez‐Escamilla & Segura‐Pérez, 2020; Richter et al., 2017; World Health Organization et al., 2018). However, that is not a reality for millions of children worldwide. In low‐ and middle‐ income countries, 250 million children are at risk of not reaching their full potential due to a variety of unfavourable conditions that threaten early childhood development (ECD), such as extreme poverty, hunger, and violence (Black et al., 2017; Clark et al., 2020; Lu et al., 2016; Richter et al., 2017; World Health Organization et al., 2018). In this context, investing in ECD services, programmes, and policies is one of the most cost‐effective mechanisms to support human development and provide an enabling environment for NC (Clark et al., 2020; World Health Organization et al., 2018).

Recently, a major effort to operationalize NC for ECD outlined five strategic actions (World Health Organization et al., 2019). Specifically, the fourth strategic action calls for countries to develop mechanisms to monitor activities that support NC across the five domains (good health, adequate nutrition, responsive caregiving, security and safety, and opportunities for early learning) at the individual, population, and programing/system level (World Health Organization et al., 2019). Building on this call, monitoring NC framework and its components have become a priority globally to support decision‐making, advocacy, and tracking progress on different governance levels (Black et al., 2017; Clark et al., 2020; Lu et al., 2020; Pérez‐Escamilla & Segura‐Pérez, 2020; Richter et al., 2017; World Health Organization et al., 2018).

Several studies have attempted to develop population‐based approaches for monitoring NC over the past few years. In this manuscript, NC population‐based approaches are defined as a set of existing indicators or a composite index methodology that uses secondary data to measure, characterize, classify, and evaluate NC environment(s) for ECD (Buccini, Coelho, et al., 2021; Köhler, 2016; UNICEF & Countdown to 2030, 2020). The Countdown to 2030 early childhood country profile, the State of Babies in the United States, and the Early Childhood Friendly Municipal Index (IMAPI) in Brazil are examples of initiatives that measure NC at the countries, state, and municipal level, respectively (Buccini, Coelho, et al., 2021; Buccini, Pedroso, et al., 2021; Keating, Cole, & Schaffner, 2020; UNICEF & Countdown to 2030, 2020). However, to our knowledge, no systematic mapping of these NC population‐based approaches has been conducted. Therefore, the aim of this scoping review is to analyse the population‐based approaches for monitoring the domains of the NC framework.

## 2 METHODS

The scoping review protocol was registered with the Open Science Framework (OSF) on August 20, 2020 (osf.io/3vxt9). We followed the PRISMA Extension for Scoping Reviews (PRISMA‐ScR) and the Joanna Briggs Institute (JBI) Manual for Evidence Synthesis for conducting the review and reporting the results (Peters et al., 2020; Tricco et al., 2018).

### 2.1 Search strategy

Searches were conducted on 25 August 2020, and the following sources were used to identify studies: (1) Databases: PubMed and Virtual Health Library (VHL); (2) Websites: United Nations Children's Fund (UNICEF), World Bank, World Bank eLibrary, World Health Organization (WHO), Grand Challenges Bill & Melinda Gates, Grand Challenges Canada, United States Agency for International Development (USAID), Organization for Economic Co‐operation and Development (OECD), Inter‐American Development Bank (IDB), Pan American Health Organization (PAHO), and Save the Children; (3) Consultation with experts on ECD (including RPE, GB, and MBG). The search results from the databases were exported into Mendeley and duplicates were removed. Additional manual searches of the reference lists in the included approaches after full text reading were performed to identify citations that might fulfil the inclusion criteria and were not identified in the electronic searches.

The search strategy was developed following the Population, Concept, and Context (PCC) strategy (Peters et al., 2020). In this scoping review, the population was children under 5 years old, the concept consisted of population‐based approaches that evaluate the NC environment for ECD, and the context encompassed all countries, states, municipalities, or cities. Medical Subject Headings (MeSH) terms and Health Science Descriptors (DeCS) were selected to operationalize the PCC search strategy. The search strategy was designed for the PubMed database and adapted for the Virtual Health Library (VHL) database. For each website, the search strategy was adapted according to its available resources and search interface. The final search strategy was validated by a health sciences librarian and is presented as Supporting Information.

### 2.2 Eligibility criteria

We included all types of study designs, articles, reports, and websites that presented population‐based approaches which evaluated the NC environment for ECD, assessing more than one domain of the NC framework (good health, adequate nutrition, responsive caregiving, security and safety, and opportunities for early learning) (World Health Organization et al., 2018). There was no restriction regarding language and publication period, and the search included both published and unpublished (grey literature) materials. We excluded studies that (1) were unavailable to retrieve (webpage not found *n* = 9; unavailable website *n* = 5; unavailable full text *n* = 9), (2) were not related with ECD, (3) evaluated only children over five years old or children with specific characteristics (e.g., preterm, low birth weight, atypical, with pathologies, twins, foster children), (4) did not assess policy environments related to early childhood (i.e., studies evaluating neonatal ICU; orphanage; natural disasters; exposure to chemicals, metals, toxins, drugs, and alcohol; and which only evaluate indicators related to the home and family), (5) did not evaluate the NC environment for ECD, and (6) did not use indicators from secondary databases.

### 2.3 Study selection

Five review authors (JP, SEACK, POS, GFC, JLP) who were previously standardized against each other (kappa = agreement of 70%) worked in pairs to screen the titles and abstracts/executive summary independently to identify potentially relevant records. The full texts of all potentially relevant citations were retrieved and independently assessed for eligibility using the predefined inclusion and exclusion criteria. Any disagreements were solved through consensus, and if necessary, by consulting a third (JP, SEACK, POS) or a fourth reviewer with expertise in the area (GB).

### 2.4 Assessment of population‐based approaches characteristics and data extraction

A pretest of the standardized protocol to extract data was done by two reviewers (JP and SEACK). First, the approaches were classified into two groups: (1) Set of indicators, which are a group of individual NC indicators (measures generated from observed facts that can evaluate performance, positions, and changes across time if evaluated regularly) that did not have a single overall summary measure and (2) composite indexes, which are a set of individual NC indicators combined into a single overall summary measure (Köhler, 2016; Organization for Economic Co‐operation and Development, 2008).

Second, the following information was extracted for all approaches: study/report reference, including approach name, authors, year of publication; aim of the approach, country, unit of analysis (country, regions, states, municipalities, and/or districts), country's economic development classification (low, middle, and/or high income), age group of children, conceptual framework if any (i.e., theoretical framework that guided the selection of indicators and/or categories), equity approach (i.e., data disaggregation or specific indicators for understanding inequity issues), number of indicators, and number of additional indicators (i.e., indicators that were used for description in the approaches and were considered supplementary by the authors, such as municipality size and socio‐economic status).

Third, for composite indexes, we also assessed methodological features, such as normalization of the indicators (i.e., standardization of indicators to allow comparisons between them), weighting of the indicators (i.e., attributing different weights to the indicators), classification of the approach's summary measure final score (e.g., country with high/medium/low performance), sensitivity or robustness analysis, and link to other indexes or indicators, which can evaluate the explanatory power of composite indexes.

Fourth, the indicators within each population‐based approach were classified into the five domains of the NC framework (good health, adequate nutrition, responsive caregiving, opportunities for early learning, and security and safety) or as demographic characteristics (World Health Organization et al., 2018). For this classification, we followed a two‐phase systematic decision tree (see Supporting Information: decision tree to classify indicators in the NC framework). In phase one, we classified the indicators of approaches that used the NC as the conceptual framework, based on the following three questions: (1.1) Are there discrepancies between approaches in the classification of indicator across the NC domains? (1.2) Are there indicators in the approaches that are not classified across the 5 NC domains? (1.3) Are there additional indicators in the approaches that can be classified into the NC domains? After that, based on the classification of the approaches that used the NC as the conceptual framework, in phase two, we classified the indicators of approaches that did not use the NC as the conceptual framework according to two questions: (2.1) Are there similar indicators in the approaches that use the NC framework as the conceptual model? (2.2) Are there additional indicators in the approaches that can be classified into the NC domains? Within each component of the NC framework, we classified the indicators into groups according to construct similarities (e.g., child mortality and child nutritional status). Fifth, given that young children's development is perceived as central to the transformation that the world seeks to achieve by 2030 and that the NC framework was developed to provide a roadmap to attain the Sustainable Development Goals (SDGs) (World Health Organization et al., 2018), we evaluated each group of indicators according to which of the 17 SDGs they cover. These classifications were validated by two senior authors with expertise in ECD (GB and MBG), and discrepancies were solved by consensus.

Sixth, we analysed if the population‐based approaches used the following format presentations: website, ranking of the country, state or municipalities scores, tables, figures, map, and profile. Based on the format presentations, we evaluated how data was shared with users. This assessment included a description of the user interaction design, including a static communication (e.g., report) that only allows users to read the presented data, or a dynamic communication, with options for users to explore the results of the approach according to their interest (e.g., interactive website that allow users to see disaggregated results by different indicators such as geographic location and municipality size). We considered that the approach was validated if the authors reported (i) that they tested the association between the set of indicators or composite index with other measures, indicators, and/or instruments, (ii) the identification of indicators was based on a participatory process involving experts on ECD and stakeholders, and/or (iii) that the indicators were selected taking into account their availability, validity, and reliability (Alexandre & Coluci, 2011; de Souza et al., 2017).

Although scoping reviews do not aim to access the quality of studies and/or approaches (Tricco et al., 2018), during data extraction, we mapped the main methodological challenges involved in building an approach to monitor the NC environment for ECD to address the gaps that could help researchers and stakeholders in using the approaches for decision‐making, as well as to improve future approaches. Therefore, we collected information on the limitations and challenges to measure the NC environment reported by the authors of each approach. Based on the quality dimensions for procedures to build and disseminate composite indicators available on the Handbook on Constructing Composite Indicators (Organization for Economic Co‐operation and Development, 2008), we conducted a qualitative analysis to identify common themes among methodological challenges and systematize gaps (i.e., information missing consensus), followed by recommended pathways to move forward. These steps were first conducted by two authors and then consensus was reached among all authors (Table 4).

## 3 RESULTS

**Figure 1:**
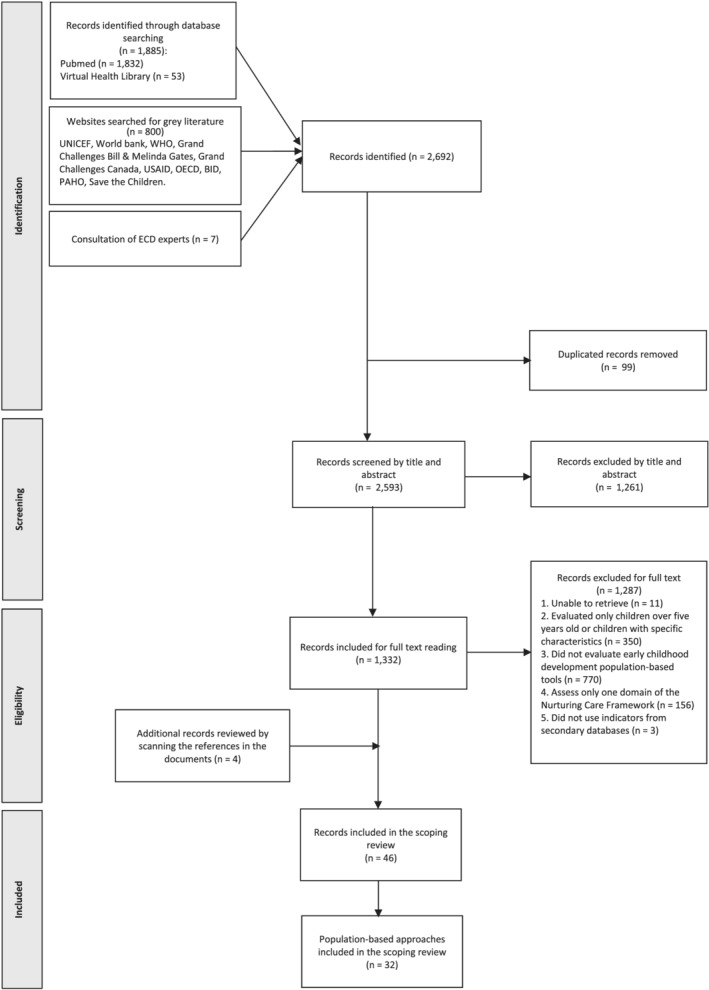
PRISMA flow diagram of the scoping review 2020

Initially, 2692 records were identified through database and website searching, and by contacting ECD experts. After the removal of duplicates, exclusions, and full text screening, 46 records were included in the scoping review. These records contained 32 different population‐based approaches for monitoring the NC environment for ECD (Figure 1). The references for each NC population‐based approach are available as Supporting Information (Matrix of references of the Nurturing Care population‐based approaches).

While the majority of approaches were composed of a set of indicators (53.1%, *n* = 17), 15 approaches were designed to estimate a composite index (46.9%). Fourteen (43.8%) approaches were launched or had their last version published between 2018 and 2020. Most approaches were applied with the country as their unit of analysis (68.8%, *n* = 22). The vast majority of approaches included middle‐income countries (84.4%, *n* = 27), and 13 included low‐income countries (40.6%). Eleven approaches were constructed to be applicable to low‐, middle‐, and high‐income countries (Table 1).

**Table 1:** Characteristics of population‐based approach for monitoring the nurturing care environment for early childhood development included in the scoping review 2020

Set of indicators vs. composite index	Population‐based approach	Authors	Year of publication	Aim	Country	Unit of analysis	Country's economic development classification	Children's age group	Conceptual framework	Equity approach	Number of indicators^a^
Set of indicators	Statistics on Newborns and Children	WHO	2010	To build a global repository for policy and health system indicators of maternal, newborn, and child health	33 countries with high under‐five mortality	Country	Middle Low	0 to 5	Not reported	Not reported	28^b^ ^,^ ^c^
Set of indicators	Doing better for children	OECD	2009	To present an overview of child well‐being and compare it across the organisation for economic co‐operation and development countries	30 countries	Country	High Middle	0 to 17	International standards agreed for children in the United Nations convention on the rights of the child	Yes	21^c^
Set of indicators	Early Childhood Development Report Card for Wealthy Countries	Save the children	2009	To evaluate how well governments are ensuring that children's earliest experiences are in the best interest of both the children and their nations' future	25 wealthy countries	Country	High Middle	Not reported	Not reported	Yes	10 and 1 additional indicator^c^
Set of indicators	Indicators of Conceptual Framework for Child Development from Birth to Age 6 (proposal)	Wu et al.	2012	To propose indicators of child development in China to identify accomplishments and understand the scope of the challenges in early child development policies	China	Country	Middle	0 to 6	Conceptual framework for child development from birth to age 6	Not reported	16 (8 measuring individual ECD outcomes; 8 measuring environments)^c^
Set of indicators	Systems Approach for Better Education Results – Early Child Development (SABER – ECD)	The World Bank	2013	To collect, synthesize, and disseminate information on ECD to enable policymakers, World Bank Group staff, and development partners to learn how countries address similar policy challenges related to ECD	39 countries	Country	High Middle Low	Not reported	SABER‐ECD framework	Not reported	42 indicators and 69 subindicators^b^ ^,^ ^c^
Set of indicators	‐	van den Heuvel et al.	2013	To highlight similarities and differences in social and health services between the countries that can inform better global ECD policies and improve early child health and development	5 countries (Sweden, Netherlands, Canada, the United States and Cuba)	Country	High Middle	Not reported	Navarro et al. conceptual framework	Not reported	17 and 12 additional indicators^c^
Set of indicators	Holistic Early Childhood Development Index (HECDI) (proposal)	UNESCO	2014	To propose a set of targets, sub targets, and indicators for the holistic monitoring of young children's well‐being at both the country and international levels	Global	Country	High Middle Low	Not reported	Holistic Early Childhood Development Index (HECDI) conceptual model and Socioecological model	Not reported	35^c^
Set of indicators	How's life for children?	OECD	2015	To promote a deeper and more engaged discussion about the changes that are needed to make children's lives better, including priorities for public policies	34 countries	Country	High Middle	0 to 17	Not reported	Yes	28^c^
Set of indicators	My childhood, my future	El‐Kogali and Krafft	2015	To offer evidence on the state of ECD in the Middle East and North Africa (MENA) to allow policy makers to implement better policies and programmes, as well as to target programmes to those with the greatest need	Middle East and North Africa (MENA), 12 countries and territories	Country	Middle	0 to 5	Not reported	Not reported	11 and 5 additional indicators^c^
Set of indicators	‐	Ford and Stein	2016	To identify scope for improvement by classifying countries across levels of risk factors for poor child development and coverage of interventions addressing these risk factors	51 countries in sub‐Saharan Africa	Country	High Middle Low	0 to 5	Not reported	Not reported	23^c^
Set of indicators	Countdown to 2030	UNICEF and WHO Countdown to 2030 Collaboration	2017 2018	To help monitor, measure, and provide recent scientific evidence on country level of women's, children's, and adolescents' health	81 countries	Country	High Middle Low	0–18	Countdown to 2030 evaluation framework	Yes	88^c^
Set of indicators	Nurturing Care indicators (proposal)	WHO, UNICEF, and World Bank Group	2018	To propose population‐based indicators for monitoring countries' progress towards sustainable development goals and child development	Global	Country	High Middle Low	Not reported	Nurturing care framework	Not reported	24^c^
Set of indicators	Australia's Children	Australian Institute of Health and Welfare Moon et al.	2020 1998	To bring together and contextualize key national statistics on child wellbeing in 1 place, providing updated data on 38 measures and supporting a more comprehensive understanding of related data gaps	Australia	Country	High	0 to 12	Australian Institute of Health and Welfare people‐centred data model and Socioecological model	Yes	38 and 10 additional indicators^c^
Set of indicators	Early Childhood First in the Municipality (*Primeira Infância Primeiro no Município*)	Maria Cecília Souto Vidigal Foundation	2020	To assess the situation of early childhood in Brazilian municipalities and provide future managers with a panel containing the most relevant indicators to prioritize children 0 to 6 years old on the government agenda	Brazil	Municipalities	Middle	0 to 6	Nurturing care framework	Yes	33^c^
Set of indicators	Early childhood inequality map 2020 (*Mapa da desigualdade da primeira infância 2020*)	Nossa São Paulo Network and Bernard van Leer Foundation Nossa São Paulo Network & Bernard van Leer Foundation	2020 2017	To strengthen the debate on inequalities in early childhood with focus on public policies that guarantee fundamental rights for children, by helping municipal stakeholders to identify priorities and needs of the population and their districts	Brazil	Districts of Sao Paulo	Middle	0 to 6	Not reported	Yes	26^c^
Set of indicators	Observatory of the Early Childhood Legal Framework (*Observatório do Marco Legal da Primeira Infância)* (especialistas)	National Early Childhood Network and Andi Communication and Rights	2020	To support the focus on the processes of formulation, implementation, monitoring, and evaluation of public policies, to ensure priority of this agenda at the local and national level	Brazil	Country, regions, states, municipalities	Middle	0 to 6	Not reported	Yes	44^c^
Set of indicators	Country profiles for early childhood development	UNICEF and the Countdown to 2030 for Women's, Children's, and Adolescents' health	2020	To establish a global monitoring and accountability system for early childhood development (ECD), attempting to compile, in one place, the available data for country and cross‐country monitoring and to provide a baseline against which progress can be monitored	197 countries	Country	High Middle Low	0 to 5	Nurturing care framework	Not reported	45^c^
Composite index	Child Development Index (*Índice de Desenvolvimento Infantil*)	UNICEF	2001	To assess the performance of the state or municipality in the process of survival, growth, and development of their children in early childhood, and contribute to the process of decentralization and municipalization of policies and services aimed at child development	Brazil	States and municipalities	Middle	0 to 6	Human rights	Yes	6^c^
Composite index	Index of Child Well‐Being in the European Union	Bradshaw et al.	2007	To compare the performance of European Union Member States and provide a picture of children's overall well‐being in the European Union	25 countries members of European Union	Country	High Middle	0 to 19	Rights of the child	Yes	51^c^
Composite index	School Success Index for Developing Countries	Save the Children	2009	To assess how well prepared young children are to succeed in school in developing countries	100 developing countries	Country	High Middle Low	0 to 5	Not reported	Not reported	6^c^
Composite index	School Success Index for the United States	Save the Children	2009	To assess how well prepared young children are to succeed in school in the United States	United States	States and district of Columbia	High	Not reported	Not reported	Not reported	5^c^
Composite index	Children's index (part of the Complete Mother's Index 2012)	Save the Children	2012a, 2012b	To document conditions for children in 165 countries and show where children fare best and where they face the greatest hardships	165 countries	Country	High Middle Low	Not reported	Not reported	Yes	3 to 5^c^
Composite index	São Paulo Early Childhood Index (*Índice Paulista da Primeira Infância*)	State System of Data Analysis Foundation and São Paulo State Government Planning and Management Department		To reflect the capacity of municipalities in the State of São Paulo to promote child development through access to health and education services for children under six years old	Brazil	Municipalities of Sao Paulo	Middle	0 to 6	Not reported	Not reported	8 and 11 additional indicators^c^
Composite index	Child Development Index	Save the Children Save the Children	2016 2008	To monitor how countries are performing in relation to the wellbeing of their children, providing an indication of the worst country in the world to be a child	94 countries (Africa, Asia, Latin America)	Country	Middle Low	0 to 24	Not reported	Yes	4^c^
Composite index	Sustainable Child Development Index (SCDI)	Chang et al.	2018a, 2018b	To evaluate countries' status of sustainable development by considering children as the key stakeholder and addressing topics in the context of inter‐generational equity (environmental, economic, and social dimensions)	138 countries	Country	High Middle Low	0 to 18	Sustainable Child Development index (SCDI) framework	Not reported	25^c^
Composite index	‐	Urke et al.	2018	To create age‐specific NC summary indexes (0–5, 6–11, and 12–23 months) suitable for research in low‐ and middle‐income countries and examine the relationship of NC to maternal resources	Colombia	Country	Middle	0 to 23 months	Socioecological model that explicitly focuses on nurturing care	Not reported	17 variables for the 0–5 months index, 18 variables for the 6–11 and 12–23 months indexes and 9 additional indicators^c^
Composite index	End of Childhood State Ranking	Save the Children	2018	To explore protection of childhood among U.S. states, focusing on some rights or guarantees of childhood: Life, healthy growth and development, education, and protection from harm	United States	States	High	0 to 19	Not reported	Not reported	5^c^
Composite index	Child Health Index	Köhler and Eriksson	2018	To distil and focus the abundant mass of data available on the national level, to be able to use them as an approach for decision makers and professionals to monitor children's health and well‐being in the 290 municipalities	Sweden	Municipalities	High	0 to 17	Not reported	Not reported	13 and 3 additional indicators^c^
Composite index	End of childhood index	Save the Children	2019	To identify the places where childhood is most preserved or most affected, based on a set of indicators related to life‐changing events that signify childhood disruption	176 countries	Country	High Middle Low	0 to 19	Not reported	Not reported	8^c^
Composite index	IMAPI ‐ Brazilian Early Childhood Friendly Municipal Index (*Índice Município Amigo da Primeira Infância*)	Buccini, Coelho, et al. Buccini, Pedroso, et al.	2021 2021	To assess the performance of the 5,570 Brazilian municipalities in relation to the provision of an enabling environment for Early Childhood Development, monitoring and identifying opportunities to support the decision making of local stakeholders	Brazil	Municipalities	Middle	0 to 5	Nurturing care framework and socioecological model	Not reported	31 indicators and 13 additional indicators^c^
Composite index	State of babies	Keating et al.	2020	To identify indicators that help advocates and policymakers compare their state's progress for infants and toddlers with that of other states	United States	Country and states	High	0 to 3	Zero to three's policy framework including good health, strong families, and positive early learning experiences	Yes	56 and 7 additional indicators^c^
Composite index	Child flourishing index	Clark et al.	2020	To construct a new national profile to measure the foundational conditions for children 0–18 years old to survive and thrive today	180 countries	Country	High Middle Low	0 to 18	UNICEF's Five Dimensions of Children's Rights in the SDGs, UN Global Strategy for Women and Children's Health, Nussbaum (2013), VanderWeele (2017), Pollard and Lee (2003) Lippman et al. (2011)	Yes	17^c^

^a^ Primary.

^b^ Primary.

^c^ Secondary.

Abbreviations: IDB, Inter‐American Development Bank; OECD, Organisation for Economic Co‐operation and Development; UNESCO, United Nations Educational, Scientific and Cultural Organization; UNICEF, United Nations Children's Fund; WHO, World Health Organization.

**Figure 2:**
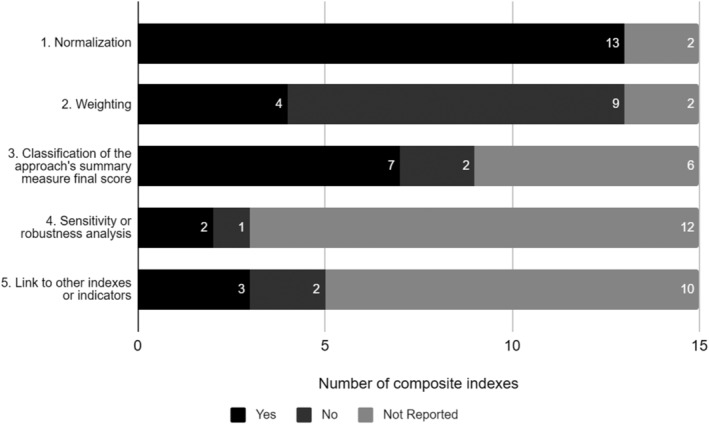
Methodological features used by the composite indexes included in the scoping review (*n* = 15) 2020

The age group of children varied across approaches, with a higher number of approaches evaluating young children (0 to 6 years old) (46.9%, *n* = 15). Half of the approaches used a conceptual framework (53.1%, *n* = 17), of which four used the NC framework (12.5%), and fourteen approaches applied an equity lens to their data (43.8%) (Table 1). The equity lens was considered to have been applied if indicators or scores were disaggregated by characteristics such as age, gender, socio‐economic status, family composition and migrant status. Among the composite indexes, most used normalization of the indicators (86.7%, *n* = 13), but only 26.7% attributed different weights to the indicators (*n* = 4). Almost half of the composite indexes classified the final score of the summary measure (46.7%, *n* = 7), only two approaches conducted sensitivity or robustness analysis, and only three were linked to other indexes or indicators (Figure 2).

**Figure 3:**
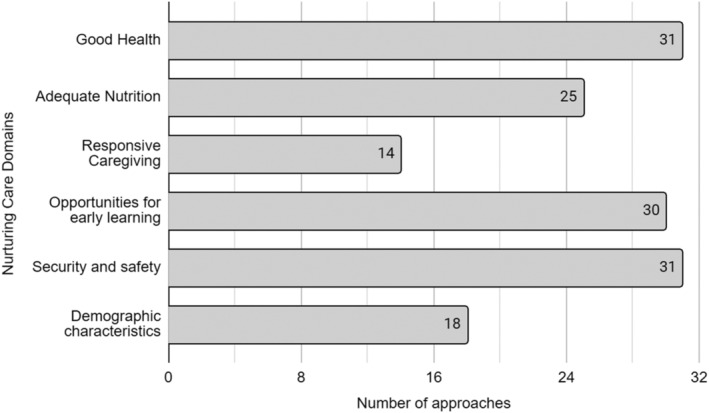
Domains of the nurturing care framework evaluated in the population‐based approaches included in the scoping review 2020

Regarding NC domains included in the approaches, while almost all the approaches included indicators within the health (96.9%, *n* = 31), security and safety (96.9%, *n* = 31), and early learning domains (93.8%, *n* = 30), more than half of the approaches did not include indicators in the responsive caregiving domain (56.3%, *n* = 18) (Figure 3). The inclusion of NC domains was different in the composite indexes than the set of indicators. While 58.8% and 88.2% of the set of indicators included the responsive caregiving and nutrition domains, respectively, only 26.7% and 66.7% of the composite indexes included theses domains.

All NC indicators (*n* = 867) were sorted into 100 groups of indicators according to their similarity across the NC framework. The groups of indicators presented in at least half of the approaches were child mortality (*n* = 24), birth weight (*n* = 18), child vaccination/immunization (*n* = 18), child nutritional status (*n* = 19), violence (*n* = 16), and vulnerability (*n* = 16) (Table 2). The SDGs covered in most groups of indicators were 3 – Good Health and Well‐Being (44.0%, *n* = 44), 16 – Peace, Justice and Strong Institutions (22.0%, *n* = 22), and 10 – Reduced Inequalities (21.0%, *n* = 21) (Supporting information: SDGs covered).

**Table 2:** Groups of indicators within each population‐based approach for monitoring the nurturing care environment for early childhood development 2020

Domain	Group (*n* = indicators)	Set of indicators																	Composite indexes														
		Statistics on newborns and children	Doing better for children	ECD report card for wealthy countries	Indicators of conceptual framework for CD from birth to age 6	SABER	van den Heuvel et al. (2013)	HECDI	How's life for children?	My childhood, my future	Ford and Stein (2016)	Countdown	Nurturing care indicators	Australia's children	EC first in the municipality	EC inequality map 2020	Observatory of the EC legal framework	Country profiles for ECD	CDI (UNICEF)	Index of child well‐being in the EU	School success index for developing countries	School success index for the US	Children's index	São Paulo EC index	CDI (Save the Children)	SCDI	Urke et al. (2018)	End of childhood state ranking	Child health index	End of childhood index	IMAPI	State of babies	Child flourishing index
Good health	Child mortality (*n* = 41)^a^	●	●				●	●	●	●		●	●	●	●	●	●	●		●	●		●	●	●	●		●		●	●	●	●
	Suicide mortality (*n* = 3)		●																							●							●
	Birth weight (*n* = 19)^a^		●		●		●	●	●		●	●		●	●	●		●		●				●		●			●		●	●	●
	Prenatal care (*n* = 14)							●		●		●	●		●	●	●	●	●												●	●	
	Delivery conditions (*n* = 14)						●	●		●		●				●	●							●							●		●
	Adolescent pregnancy (*n* = 17)		●						●			●	●	●	●	●		●		●						●		●	●	●	●		●
	HIV (*n* = 6)										●	●					●	●															
	Child vaccination/immunization (*n* = 29)^a^	●	●		●		●	●		●		●	●	●			●		●	●						●	●		●		●	●	●
	Diarrhoea treatment (*n* = 9)	●						●				●	●																				
	Respiratory diseases (*n* = 11)	●						●				●	●			●		●													●		
	Preterm (*n* = 3)																	●													●	●	
	Congenital syphilis (*n* = 3)															●	●														●		
	Postnatal care (*n* = 5)											●	●					●													●		
	Coverage of health services (*n* = 7)						●	●					●		●		●														●		
	Other diseases or conditions (*n* = 14)													●						●						●	●		●				
	Adolescent mortality (*n* = 3)								●			●								●													
	Malaria infection (*n* = 5)							●			●	●																					
	Health behaviour (*n* = 6)		●						●					●						●													
	Maternal mortality (*n* = 12)											●	●				●	●													●	●	●
	Well‐being of child and young people (*n* = 9)								●											●													
	Policies, laws, programmes, and action for newborn, child, and maternal health (*n* = 14)	●				●		●				●																				●	
	Family planning (*n* = 4)											●																					
	Access and quality of health facilities (*n* = 17)	●		●		●						●				●	●														●	●	
	Expenditure on healthcare (*n* = 8)	●					●					●														●							
Adequate nutrition	Child nutritional status (*n* = 29)^a^	●			●			●	●	●	●	●	●	●	●		●	●		●			●		●					●	●	●	●
	Women nutritional status (*n* = 5)				●							●																					
	Breastfeeding (*n* = 19)	●	●				●				●	●	●	●	●			●									●		●			●	
	Diet characteristics (*n* = 10)										●	●	●					●		●							●				●		
	Anaemia (*n* = 4)										●	●	●																				
	Food security (*n* = 3)																											●			●	●	
	Micronutrient supplementation (*n* = 4)				●							●																				●	
	Food fortification (*n* = 4)									●	●	●																					
	Lifetime cost of growth deficit in early childhood (*n* = 1)																	●															
	Policies, laws, and programmes that support adequate nutrition (*n* = 8)	●				●						●						●													●		
Responsive caregiving	Development milestones (*n* = 19)				●			●					●	●				●														●	
	Inadequate supervision (*n* = 6)							●			●		●					●									●						
	Well‐being of caregivers (*n* = 8)							●			●			●				●				●					●					●	
	Home visits programmes (*n* = 6)														●		●	●													●	●	
	Programmes, services, and strategy for ECD (*n* = 16)					●												●									●					●	
	Family resilience (*n* = 1)																															●	
	Social support (*n* = 2)													●																			
	Child development screening (*n* = 3)					●																										●	
	Recognizing and responding to illness and danger signs (*n* = 1)	●																															
Opportunities for early learning	Caregiver‐child interactions (*n* = 14)								●	●				●				●		●		●					●					●	
	Access to educational supplies (*n* = 8)										●		●					●		●													
	Access to education and enrolment (*n* = 50)			●		●		●		●					●	●	●		●	●	●	●	●	●		●					●		
	Educational deprivation (*n* = 2)		●						●																								
	School characteristics (*n* = 5)														●		●	●															
	School attendance (*n* = 9)				●						●			●	●		●	●											●				
	Training or qualification of childcare staff (*n* = 12)			●		●		●									●							●							●	●	
	Child care staff proportion (*n* = 8)			●				●								●								●							●	●	
	School environment (*n* = 6)					●											●														●		
	Child educational performance (*n* = 21)		●				●	●	●		●			●						●	●	●						●					●
	Child feelings about school (*n* = 6)		●						●											●													
	Out‐of‐school children (*n* = 6)								●											●	●				●				●	●			
	Support to education (*n* = 15)			●		●	●	●		●				●												●						●	
Security and safety	Violence (*n* = 23)^a^							●	●	●			●	●	●	●		●		●						●	●	●		●	●	●	●
	Birth registration (*n* = 8)					●		●				●	●				●	●							●								
	Sanitation (*n* = 18)							●				●	●			●	●	●					●				●				●		●
	Cash transfer programmes (*n* = 5)														●		●														●	●	
	Environment (*n* = 4)																									●					●		
	Alcohol and smoking (*n* = 12)		●				●							●						●						●			●				
	Drugs/substance use (*n* = 2)																			●													
	Insecticide treated nets and spraying (*n* = 5)	●									●	●																					
	Bullying (*n* = 4)		●						●					●						●													
	Child labour (*n* = 2)									●																				●			
	Home environment (*n* = 13)		●						●					●		●				●												●	
	Neighbourhood environment (*n* = 9)													●		●				●												●	
	Children in foster care (*n* = 6)													●			●															●	
	Child or adolescent marriage (*n* = 3)										●																			●			●
	Homelessness (*n* = 1)													●																			
	Traffic accidents (*n* = 2)															●																	●
	Access to social assistance services (*n* = 1)																●																
	Electricity (*n* = 1)																									●							
	Child and adolescent sexual behaviour (*n* = 2)																			●													
	Hygiene habits (*n* = 1)																										●						
	Insurance coverage (*n* = 1)																															●	
	Injuries or mortality due to environmental factors (*n* = 3)													●												●			●				
	Vulnerability (*n* = 27)^a^		●	●				●	●				●	●	●	●	●	●		●				●					●		●	●	●
	Health insurance (*n* = 7)																															●	
	Discipline (*n* = 1)																	●															
	Family structure (*n* = 5)																			●		●										●	
	Immigrants and refugees (*n* = 3)						●							●																			
	Adult education (*n* = 8)							●			●	●							●		●						●						
	Adult unemployment (*n* = 4)								●											●						●							
	Adverse Childhood Experiences (ACEs) (*n* = 2)																															●	
	Parental leave (*n* = 6)			●				●										●														●	
	Policies, laws, and programmes for social protection (*n* = 18)			●		●		●				●			●			●														●	
	Policy governance (*n* = 3)					●																											
Demographic characteristics	Fertility rate (*n* = 2)											●									●												
	Births (*n* = 4)											●						●									●				●		
	Child and adolescent population (*n* = 21)	●								●		●		●	●	●	●	●						●					●		●	●	
	Total population (*n* = 9)						●			●		●			●	●		●						●							●		
	Geographic scope (*n* = 6)	●																													●	●	
	Age and sex of child or mother (*n* = 5)													●												●	●						
	Race/ethnicity (*n* = 1)																															●	
	Socio‐economic indicators (indexes) (*n* = 17)						●			●								●						●		●	●					●	
	Maternal occupation (*n* = 2)																										●					●	
	Politics and civic participation (*n* = 5)						●		●											●													
	Life expectancy (*n* = 1)									●																							
Total (*n* = 867)	Total number of groups (*n* = 100)																																

Abbreviations: CD, Child Development; CDI, Child Development Index; EC, Early Childhood; ECD, Early Childhood Development; EU, European Union; HECDI, Holistic Early Childhood Development Index; IMAPI, Early Childhood Friendly Municipal Index (*Índice Município Amigo da Primeira Infância*); SABER, Systems Approach for Better Education Results; US, United States; SCDI, Sustainable Child Development Index.

^a^ Half or more of the approaches have some indicator in the groups: Child mortality, Birth weight, Child vaccination/immunization, Child Nutritional Status, Violence, and Vulnerability.

To disseminate the approaches, less than a half had websites (25.0%, *n* = 8), presented rankings (43.8%, *n* = 14), or maps (21.9%, *n* = 7). To share the population‐based approaches with the end users (e.g., decision makers), less than one fourth (21.9%, *n* = 7) had a dynamic type of data communication, and the type of user interaction design was similar between composite indexes and set of indicators. Twenty of the 32 approaches included were validated (62.5%). While less than one third (29.4%, *n* = 5) of the set of indicators have been validated, 80% (*n* = 12) of the composite indexes have been validated (Table 3). The following criteria used to consider the approaches valid was (i) the indicator selection process prioritizes those that are valid, reliable and up‐to‐date, for example, the Child Development Index; (ii) built based on participatory process, for example, the Early Childhood Legal Framework; (iii) tested the association between the results of the approach and other indicators, that is, Early Childhood Friendly Municipal Index (IMAPI), which confirmed associations of the index scores with school achievement and children's social vulnerability indicators.

**Table 3:** Data presentation format used by each population‐based approach for monitoring the nurturing care environment for early childhood development 2020

Set of indicators vs. composite index	Population‐based approach	Data presentation resources							User interaction design		Validity
		Website	Ranking	Tables	Figures	Map	Profile	Not applicable	Static	Dynamic	
Set of indicators	Statistics on newborns and children			√					√		√^a^
Set of indicators	Doing better for children		√	√	√		√		√		
Set of indicators	Early childhood development report card for wealthy countries		√	√					√		NA
Set of indicators	Indicators of conceptual framework for child development from birth to age 6 (proposal)							√	NA		
Set of indicators	Systems Approach for Better Education Results – Early Child Development (SABER – ECD)	√			√		√		√	√	
Set of indicators	van den Heuvel et al. (2013)			√					√		NA
Set of indicators	Holistic Early Childhood Development Index (HECDI) (proposal)							√	NA		
Set of indicators	How's life for children?				√				√		
Set of indicators	My childhood, my future			√	√				√		
Set of indicator	Ford and Stein (2016)			√		√			√		√^a^ ^,^ ^b^
Set of indicators	Countdown to 2030	√				√	√		√	√	NA
Set of indicators	Nurturing care indicators (proposal)							√	NA		
Set of indicators	Australia's Children				√				√		
Set of indicators	Early Childhood First in the Municipality (*Primeira Infância Primeiro no Município*)	√			√	√	√			√	
Set of indicators	Early childhood inequality map 2020 (*Mapa da desigualdade da primeira infância 2020*)			√		√			√		√^a^
Set of indicators	Observatory of the Early Childhood Legal Framework (*Observatório do Marco Legal da Primeira Infância)*	√		√	√		√			√	√^a^ ^,^ ^c^
Set of indicators	Country profiles for early childhood development	√					√				√^a^
Composite index	Child Development Index (*Índice de Desenvolvimento Infantil*)		√	√	√				√		√^a^
Composite index	Index of Child Well‐Being in the European Union		√	√	√				√		√^a^
Composite index	School Success Index for Developing countries		√	√					√		√^a^
Composite index	School Success Index for the United States		√	√					√		√^a^
Composite index	Children's index (part of the Complete Mother's Index 2012)		√	√					√		√^a^
Composite index	São Paulo Early Childhood Index (*Índice Paulista da Primeira Infância*)	√		√	√	√	√			√	√^a^
Composite index	Child Development Index		√		√				√		√^a^ ^,^ ^b^
Composite index	Sustainable Child Development Index (SCDI)		√	√		√			√		√^b^
Composite index	Urke et al. (2018)			√					√		√^a^
Composite index	End of Childhood State Ranking		√	√					√		√^a^
Composite index	Child Health Index			√	√				√		√^a^
Composite index	End of childhood index		√	√	√				√		√^b^
Composite index	IMAPI ‐ Brazilian Early Childhood Friendly Municipal Index (*Índice Município Amigo da Primeira Infância* )	√	√				√			√	√^a^ ^,^ ^c^
Composite index	State of babies	√	√			√	√			√	√^a^ ^,^ ^b^ ^,^ ^c^
Composite index	Child flourishing index		√	√	√				√		√^a^

*Note:* Static type of data communication: allows only reading of the presented data; Dynamic type of communication: provides options for the user to “navigate” or to explore the results of the approach.

Abbreviation: NA, not applicable.

^a^ For this approach, it was reported that the indicators were selected based on its availability, validity, and reliability.

^b^ For this approach, it was reported that association tests between the set of indicators or composite index were conducted with other measures, indicators, and/or instruments.

^c^ For this approach, it was reported that the identification of indicators was based on a participatory process involving experts on ECD and stakeholders.

We identified six methodological challenges regarding the construction of NC monitoring approaches. While well‐known methodological challenges related to the lack of clarity about the definition of NC indicators and data quality and availability were mapped, we also identified challenges about adding an equity lens to the definition of NC domains and operationalization of validity of the approaches, weighting of indicators, and forms of sharing data with the end users (Table 4).

**Table 4:** Pathways to move forward with the methodological challenges of building and using an approach to monitor Nurturing Care environment for Early Childhood Development

Methodological challenges	Gap	Pathways to move forward
Lack of clarity about the definition of nurturing care indicators	There is no international consensus on the best indicators to measure the nurturing care environment, posing a challenge for researchers and stakeholders on the best indicators to adequately measure the complexity of the environment.	Agree on a common approach to select indicators that are relevant and suitable to different contexts (e.g., guidance on participatory approaches). Develop a guidance on basic indicators that needs to be prioritized and others that can be adapted to each sociocultural and political context. The Nurturing Care Framework is a good theoretical framework for the construction of this common base.
Data quality and availability	Critical aspects are related to timeliness and completeness of data, comparability between units of analysis (countries/states/municipalities), use of proxy measures for some indicators, self‐report survey data, and data accuracy. This aspect was the most reported limitation and challenge when building an approach.	Prioritize standardized data collected from routine information systems and complement with data from recurrent surveys. Use data from internationally recognized institutions.
Equity approach	Lack of equity lens when building a nurturing care monitoring system (i.e., consideration of gender, ethnicity, social class, and other social characteristics on the indicators and/or approaches).	Consider multiple layers of equity aspects when selecting nurturing care indicators. Advocate for data collection that provides disaggregated data on these aspects.
Validity of approaches	Validation is essential to ensure that the assessment approach is truthful and accurate in measuring the nurturing care. Assessing construct validity was identified as the greatest challenge in this regard.	Develop processes that allows building the approach in a participatory way (government, researchers, international institutions). Define other indicators and/or composite indexes related to child development outputs and inputs to test the correlation with the results of the approach.
Weighing of indicators (only for composite indexes)	Whether or not to weigh indicators of composite index is not a consensus. It may also be due to the lack of a solid empirical or statistical basis in the construction of the composite approaches so far.	Starting from a strong theoretical base that enables the understanding of causal relationships. Conduct a participatory process with experts to identify the best way to weigh the indicators. Test different statistical methods of weighing method and define the best fit based on clear and transparent criteria
Sharing of nurturing care data to users	Type of resources used to communicate data may limit the possibilities for understanding and analysing the results.	Conduct small meetings with key stakeholders to consult with them about the best forms to provide data for their decision making. Develop dynamic resources for data communication, such as interactive websites, maps, etc. Provide a manual with clear steps on how the approach was built, which data were used, and the aspects of validity can help increasing the use of data.

## 4 DISCUSSION

We mapped 32 population‐based approaches for monitoring the NC environment for ECD. We noted an increase in the reporting of approaches after 2018, coinciding with the launch of the NC framework, which is the major global conceptual framework to advance science and policy making related to ECD (World Health Organization, 2019; World Health Organization et al., 2018). The growing number of population‐based approaches across different contexts observed in our review clearly respond to the call for countries to monitor ECD through relevant NC indicators to inform integrated ECD policies, programmes, and services, which is important especially for advancing advocacy and effective decision‐making (Köhler, 2016; World Health Organization et al., 2018). Given the need to advance the NC, our scoping review gathered, systematized, and analysed the population‐based approaches available in the literature to monitor and characterize the NC environment for ECD across countries regardless of their economic development level, with no language and publication period restrictions.

Approximately one third of the population‐based approaches were developed simultaneously for low‐, middle‐, and high‐income countries. Especially in low‐ and middle‐ income countries (LMIC), the investment in ECD from a NC perspective is relatively new; thus, effective monitoring approaches can help to improve policymaking, advocacy, and monitoring progress in integrated ECD (Britto et al., 2016). While comparability and exchange across countries is important to guide international advocacy and collaborations towards an enabling NC environment for ECD, our findings indicate major differences in the indicators and methodologies informing each population‐based approach, which does not facilitate comparisons or foster collaborations.

One of the approaches mapped in this scoping review and led by the WHO, UNICEF, and the World Bank Group recommends that countries have a small set of core global indicators that are relevant and standardized (e.g., indicators from SDGs and the Global Strategy for Women's, Children's, and Adolescents' Health monitoring), in addition to country specific indicators that are relevant for each country's priorities (World Health Organization, 2019; World Health Organization et al., 2018). However, that is not the reality identified in our scoping review. Rather, an extremely large number of different indicators have been used across approaches. Even when the indicators are grouped for similarity this number is still high (*n* = 100 groups of indicators). Child mortality, birth weight and vaccination/immunization, infant nutritional status, violence, and vulnerability were found in at least half of the approaches and may serve as a starting point to define a small set of indicators to be compared across settings. In this direction, primary surveys of countries can standardize the data collection of this small set of indicators for global monitoring, allowing the comparison of results between countries.

The creation of approaches to monitor the NC environment can be a powerful instrument for decision‐making (World Health Organization, 2019); however, the creation of several approaches with the same goal can make it difficult for stakeholders to prioritize approaches for decision‐making. In Brazil, for example, four approaches were launched with similar aims all in 2020 (Buccini, Coelho, et al., 2021; Buccini, Pedroso, et al., 2021; Maria Cecília Souto Vidigal Foundation, 2020; National Early Childhood Network & Andi Communication and Rights, 2020; Nossa São Paulo Network & Bernard van Leer Foundation, 2020), and only one has been fully validated (Buccini, Coelho, et al., 2021; Buccini, Pedroso, et al., 2021). Therefore, due the amount of time and resources required to develop approaches for monitoring NC environments, we recommend that countries prioritize the development of valid monitoring approaches or invest in validating existing approaches.

It is well‐known that children can experience different social inequalities within a region of a country, a state, or a municipality (Buccini, Coelho, et al., 2021; Buccini, Pedroso, et al., 2021; Clark et al., 2020; Keating, Murphey, et al., 2020; São Paulo for Early Childhood et al., 2015). For that reason, approaches that measure the NC at the local levels and take an equity approach are necessary to guide the development of equitable programmes and systems for the most vulnerable populations (Clark et al., 2020; World Health Organization et al., 2018). However, the majority of approaches mapped in our scoping review did not have disaggregation by geographic location or an equity approach. A shift to more local level monitoring approaches is expected as we move forward, especially in LMIC given the need to understand where the vulnerabilities are. Therefore, we recommend that approaches include indicator(s) that can be disaggregated by geographic location, sociodemographic (i.e., gender and ethnicity), and economic characteristics (i.e., social status), to allow the identification of the most vulnerable families (Clark et al., 2020; World Health Organization et al., 2018).

In addition, decision‐makers must understand how NC environments change over time. To allow timely tracking of progress, our findings support the call to include NC indicators in routine information systems, and not only indicators from cross‐sectional population‐based surveys (World Health Organization, 2019; World Health Organization et al., 2018, 2019). An example of an innovative NC environment monitoring approach that uses routine information systems is the Brazilian ECD Friendly Municipal Index (IMAPI) reported in this supplement issue (Buccini, Coelho, et al., 2021; Buccini, Pedroso, et al., 2021). IMAPI illustrates the central role that governments should have in providing complete and updated user‐friendly information to guide improvements in coverage and quality of NC environments. This effort requires investments in efficient decentralized information systems with sustainable management appropriate for the country's context, in addition to the formation of human resources to facilitate their use. Therefore, data availability and data quality from routine information systems must be available; however, this is not the reality in many countries (World Health Organization, 2019). When data of interest are not available, proxy measures (e.g., school success index for developing countries uses gross intake ratio in the last grade of primary school as a proxy for primary school completion) can be used, and the data quality should always be prioritized by the selection of relevant, accurate, and coherent indicators (Organization for Economic Co‐operation and Development, 2008; Save the Children, 2009).

The use of sound conceptual models, such as the NC framework (World Health Organization et al., 2018), can support an intersectoral approach to improve NC and can also facilitate comparisons across countries and the efficient identification of data gaps for decision making. There is a lack of responsive caregiving indicators across countries (Richter et al., 2020; UNICEF & Countdown to 2030, 2020; World Health Organization, 2019). Richter et al. (2020) highlight the importance of an operational definition for responsive caregiving to measure and compare it across different scenarios (Richter et al., 2020). In addition, our findings support the recommendation that countries include responsive caregiving indicators in their nationally representative surveys and routine monitoring system, such as caregiver's mental health, parental support groups, and home visits (UNICEF & Countdown to 2030, 2020; World Health Organization, 2019), because these indicators will be relevant to monitor ECD over time and to improve targeting of public policies and programmes.

There are trade‐offs between population‐based approaches composed of a set of indicators (i.e., a group of individual NC indicators without a single overall summary measure) and those composite indexes (i.e., NC indicators combined into a single overall summary measure); thus, the choice of one over the other should be based on the goals and intended use (Köhler, 2016; Organization for Economic Co‐operation and Development, 2008). This scoping review found that composite indexes were more likely to be validated, while the sets of indicators were more likely to include indicators from responsive caregiving and adequate nutrition domains. In addition, the sets of indicators have the advantage of providing detailed information of each NC indicator and domain included, but the disadvantage of being more complex and difficult to interpret, especially in the context of measuring a complex concept as the NC, because separated indicators require the ability to identify common trends across the multidimensional aspects covered in each approach (Organization for Economic Co‐operation and Development, 2008). On the other hand, the composite indexes summarize into a single measure several indicators and dimensions of the NC framework. They are easy to interpret and to communicate findings but can lead to an oversimplified conclusion. For example, composite indicators usually combine very heterogeneous indicators that can have different levels of performance. Therefore, composite population‐based approaches, including IMAPI (Buccini, Coelho, et al., 2021; Buccini, Pedroso, et al., 2021) and The US ‘State of Babies’ initiative (Keating, Murphey, et al., 2020), have combined both strategies of providing a summary measure as well as the results of individual indicators. This strategy can allow stakeholders to look at the whole picture of the overall NC environments or by domain or indicator, depending on their questions.

While composite indexes can combine and summarize complex and multidimensional measures in a more accessible and useful manner for advocates and decision‐makers, they can be misleading and generate over simplified conclusions if they are not well‐constructed and correctly interpreted, for example, when there is a random selection of indicators or a combination of too disparate indicators (Köhler, 2016; Organization for Economic Co‐operation and Development, 2008). Therefore, approaches with composite indexes must be well‐designed, following carefully evidence‐based methodologies (Buccini, Coelho, et al., 2021). The indicators are often standardized to allow comparisons among them and aggregations (Köhler, 2016). The weighting of indicators in composite indexes can improve reliability and is recommended when some indicators contribute more to the outcome than others in composite indexes; nevertheless, most approaches use an equal weighting for all indicators. The methods for weighing indicators include statistical methods and methods involving experts that discuss and reach a consensus on the importance of each indicator for the outcome in question (Köhler, 2016; Organization for Economic Co‐operation and Development, 2008). For thematic fields that still lack consensus on the most appropriate way to evaluate them, the participatory process with experts can be a way to reach a more coherent result. Regardless of the method chosen, the process must be transparent and clearly reported. Best practices for building either a set of indicators or a composite index also include choosing available indicators according to their validity (i.e., ability to measure what it is intended to measure), relevance, and reliability (i.e., consistency in different situations) as well as giving preference to indicators that are already properly operationalized and conceptualized (de Souza et al., 2017; Köhler, 2016).

Delivering NC data to those who will use them in an accurate and comprehensive manner can be challenging. To be effective, data should be presented to decision makers in creative and user‐friendly formats, such as graphs, dashboards, score cards, rankings, and maps (Köhler, 2016; Organization for Economic Co‐operation and Development, 2008; World Health Organization, 2019; World Health Organization et al., 2018). The dynamic type of communication is likely to be a more useful option because it allows stakeholders to interact with data according to their interests and goals. IMAPI is an example of a population‐based approach with dynamic communication. It has a user‐friendly format, presenting on the website a page for each municipality, a ranking for the general index and for each domain of the NC framework, it also allows the exploration of data at the regional, state, and municipal scale (Buccini, Coelho, et al., 2021; Buccini, Pedroso, et al., 2021). We recommend that researchers not only focus on sharing data but also on how to improve the understanding, interest, and interpretation by stakeholders, professionals, and the overall population (Köhler, 2016; Organization for Economic Co‐operation and Development, 2008; Sarkies et al., 2017; World Health Organization et al., 2018). To make the message clearer, researchers may use knowledge‐brokers and other materials (e.g., country profile, maps) and avoid the use of technical language (Baker et al., 2018; Sarkies et al., 2017). They should also make resources available that support transformation, given that an adequate presentation of data to its users may be an important step to support decision‐making (Sarkies et al., 2017). In addition to the presentation, the approaches must be valid and reliable to support high quality decision‐making processes. Stakeholders, including government, researchers, civil society organizations, and international institutions, should participate in the development of approaches to monitor the NC, guaranteeing that the data addresses the key decision‐making related questions. Unfortunately, despite its importance, only half of the approaches have been validated; thus, efforts should be made to validate the existing approaches guaranteeing to stakeholders that they can trust the information provided to guide decision‐making.

We acknowledge that our scoping review has some limitations. As other literature reviews, the publication and selection bias may affect the results. However, to decrease the risk of this type of bias, we registered the protocol a priori and included in the search strategy grey literature and consultation with ECD experts. Despite these limitations, the findings of this scoping review are robust and elucidate a gap in the literature by mapping the characteristics of population‐based approaches for monitoring the NC environment for ECD. More studies are necessary to validate the population‐based approaches and address how the information provided by them can actually promote evidence‐based decision‐making. Thus, we recommend that future studies apply sound implementation science and research methods to design, implement, scale up, and evaluate strategies with the potential to improve the NC environment for ECD, for a better future for children. In addition, it is important that future studies verify whether and how the approaches are used by stakeholders and the quality with which they are being implemented. Qualitative studies with programme planners, policymakers, government officials, and researchers, as well as with civil society organizations and the general population will be key to evaluate implementation quality aspects of diverse NC environments monitoring systems.

Based on the results of this scoping review, we recommend that (1) a global monitoring system with a set of core NC indicators should be created, allowing comparisons across countries and throughout time (World Health Organization, 2019); (2) disaggregated data should be available, facilitating the identification of the most vulnerable groups according to geographic location, sociodemographic, and economic characteristics (Clark et al., 2020; World Health Organization et al., 2018); (3) approaches should focus on a continuous monitoring of NC indicators, allowing stakeholders to identify barriers for integrated ECD programmes and services and to promptly address them to maintain adequate levels of coverage and quality over time; (4) approaches should be systematically constructed, validated, and interpreted, based on a careful selection of robust, relevant, reliable, and valid indicators (Köhler, 2016; Organization for Economic Co‐operation and Development, 2008); (5) authors should provide specific and transparent reporting of the methodology followed (Köhler, 2016) so that others can replicate their findings; (6) countries should address the NC data gaps identified in this scoping review, especially for responsive caregiving indicators (Richter et al., 2020); and (7) approaches should be presented and disseminated in a user‐friendly dynamic format, increasing their comprehension and use by the stakeholders and the overall population (Köhler, 2016; Organization for Economic Co‐operation and Development, 2008).

In summary, the existing approaches vary greatly in their methodology for selecting indicators or composite indexes to measure the NC environment. Standardized methodologies to select sensitive indicators are needed, and they must consider data availability, data quality, and level of disaggregation. User‐friendly formats to report and disseminate valid approaches to measure NC environment(s) are necessary to support decision‐making to advance ECD globally.

## CONFLICT OF INTEREST

The authors declare that they have no conflicts of interest.

## CONTRIBUTIONS

GB, JP, SEACK, and MBG have designed the systematic review protocol with inputs of RPE. JP, SEACK, POS, GFC, JLP, and GB conducted all the steps of bibliography searches and paper selection, and also conducted the analysis and interpreted the results. JP wrote the first draft of the manuscript with intellectual input of GB. MBG, RPE, and GB revised the draft of the manuscript critically through an iterative process. All authors revised and approved the final version of the manuscript.

## Supporting information


**Figure S1.** Supporting Information

**Data S1.** Supporting Information

**Data S2.** Supporting Information

**Data S3.** Supporting Information

## Data Availability

The data that supports the findings of this study are available in the supplementary material of this article.
