# Supplementary material for: Population‐based approaches for monitoring the nurturing care environment for early childhood development: A scoping review
Source: Matern Child Nutr. 2021 Nov 4;18(Suppl 2):e13276. doi: 10.1111/mcn.13276 (PMC8968941; doi:10.1111/mcn.13276)
Supplement: Supplementary file 1 — Figure S1. Supporting Information [file MCN-18-e13276-s002.pdf]

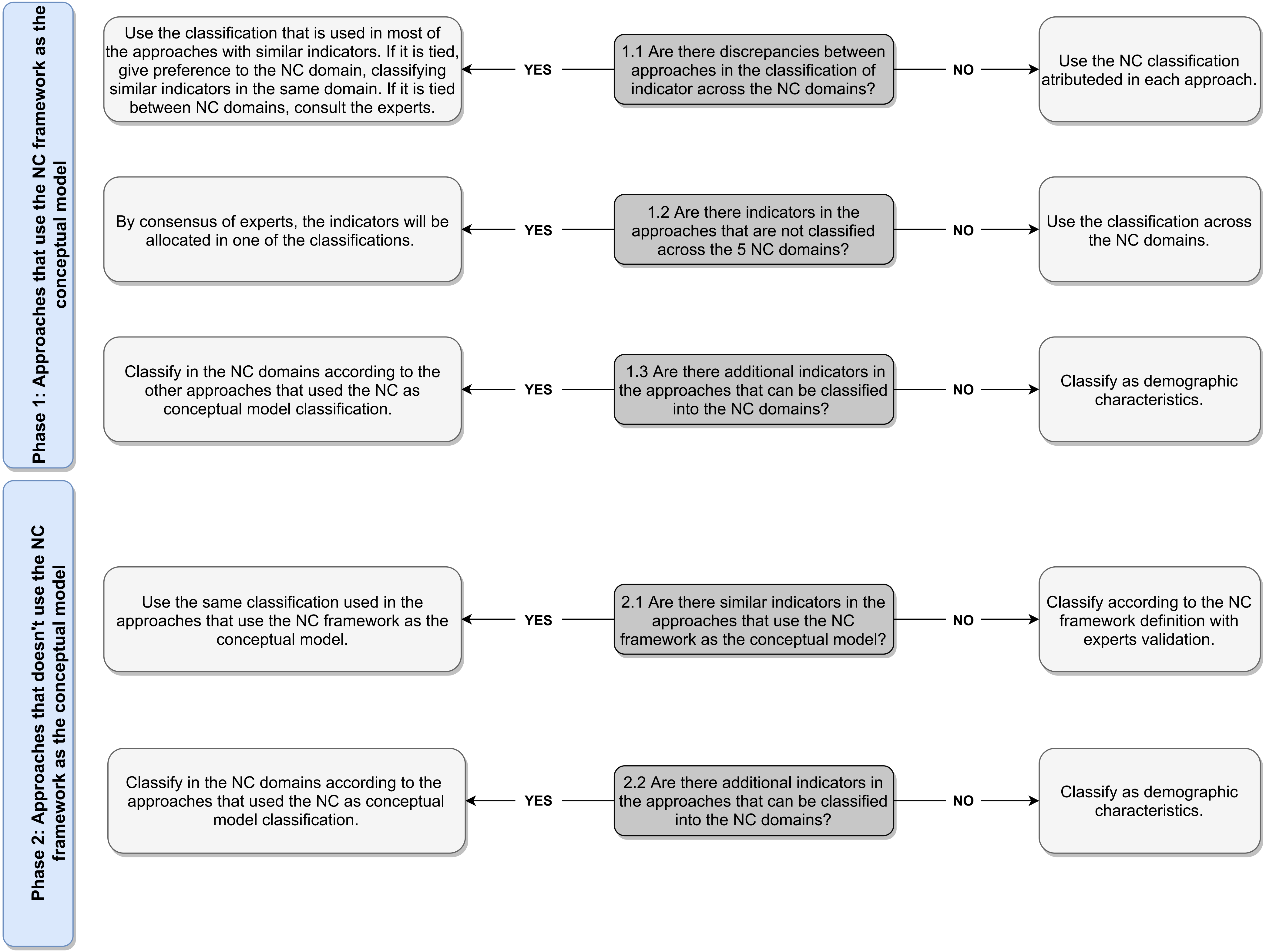
Phase 1: Approaches that use the NC framework as the conceptual model
Use the classification that is used in most of the approaches with similar indicators. If it is tied, give preference to the NC domain, classifying similar indicators in the same domain. If it is tied between NC domains, consult the experts.
YES
1.1 Are there discrepancies between approaches in the classification of indicator across the NC domains?
NO
Use the NC classification atributeded in each approach.
By consensus of experts, the indicators will be allocated in one of the classifications.
YES
1.2 Are there indicators in the approaches that are not classified across the 5 NC domains?
NO
Use the classification across the NC domains.
Classify in the NC domains according to the other approaches that used the NC as conceptual model classification.
YES
1.3 Are there additional indicators in the approaches that can be classified into the NC domains?
NO
Classify as demographic characteristics.
Phase 2: Approaches that doesn't use the NC framework as the conceptual model
Use the same classification used in the approaches that use the NC framework as the conceptual model.
YES
2.1 Are there similar indicators in the approaches that use the NC framework as the conceptual model?
NO
Classify according to the NC framework definition with experts validation.
Classify in the NC domains according to the approaches that used the NC as conceptual model classification.
YES
2.2 Are there additional indicators in the approaches that can be classified into the NC domains?
NO
Classify as demographic characteristics.
