# Supplementary material for: Population‐based approaches for monitoring the nurturing care environment for early childhood development: A scoping review
Source: Matern Child Nutr. 2021 Nov 4;18(Suppl 2):e13276. doi: 10.1111/mcn.13276 (PMC8968941; doi:10.1111/mcn.13276)
Supplement: Supplementary file 2 — Data S1. Supporting Information [file MCN-18-e13276-s004.docx]

**Supporting Information.** Matrix of references of the Nurturing Care population-based approaches.

| **Approach name** | **Reference** | **Website** |
| --- | --- | --- |
| Statistics on Newborns and Children | WHO (2010) | - |
| Doing better for children | OECD (2009) | - |
| Early Childhood Development Report Card for Wealthy Countries | Save the Children (2009) | - |
| Indicators of Conceptual Framework for Child Development from Birth to Age 6 | Wu et al. (2012) | - |
| Systems Approach for Better Education Results – Early Child Development (SABER – ECD) | The World Bank (2013) | saber.worldbank.org |
| - van den Heuvel et al. (2013) | van den Heuvel et al. (2013) | - |
| Holistic Early Childhood Development Index (HECDI) | UNESCO (2014) | - |
| How's life for children? | OECD (2015) | - |
| My childhood, my future | El-Kogali and Krafft (2015) | - |
| - Ford and Stein (2016) | Ford and Stein (2016) | - |
| Countdown to 2030 | UNICEF and WHO (2017) | countdown2030.org |
| Nurturing Care indicators | WHO, UNICEF, and World Bank Group (2018) | - |
| Australia's Children | Australian Institute of Health and Welfare (2020) | - |
| Early Childhood First in the Municipality (*Primeira Infância Primeiro no Município*) | Maria Cecília Souto Vidigal Foundation (2020) | primeirainfanciaprimeiro.fmcsv.org.br |
| Early childhood inequality map 2020 (*Mapa da desigualdade da primeira infância 2020*) | Nossa São Paulo Network and Bernard van Leer Foundation (2020) | - |
| Observatory of the Early Childhood Legal Framework (*Observatório do Marco Legal da Primeira Infância)* | National Early Childhood Network and Andi Communication and Rights (2020) | rnpiobserva.org.br |
| Country profiles for early childhood development | UNICEF and Countdown to 2030 (2020) | nurturing-care.org/resources/country-profiles/ |
| Child Development Index (*Índice de Desenvolvimento Infantil*) | UNICEF (2001) | - |
| Index of Child Well-Being in the European Union | Bradshaw et al. (2007) | - |
| School Success Index for Developing Countries | Save the Children (2009) | - |
| School Success Index for the United States | Save the Children (2009) | - |
| Children's index (part of the Complete Mother's Index 2012) | Save the Children (2012) | - |
| São Paulo Early Childhood Index (*Índice Paulista da Primeira Infância*) | State System of Data Analysis Foundation and São Paulo State Government Planning and Management Department (2015) | ippih.seade.gov.br |
| Child Development Index | Save the Children (2016) | - |
| Sustainable Child Development Index (SCDI) | Chang et al. (2018) | - |
| - Urke et al. (2018) | Urke et al. (2018) | - |
| End of Childhood State Ranking | Save the Children (2018) | - |
| Child Health Index | Köhler and Eriksson (2018) | - |
| End of childhood index | Save the Children (2019) | - |
| Early Childhood Friendly Municipal Index (*Índice Município Amigo da Primeira Infância - IMAPI*) | University of Brasilia, Federal University of Bahia, and Yale School of Public Health (2020) | imapi.org |
| State of Babies | Keating et al. (2020) | stateofbabies.org |
| Child flourishing index | Clark et al. (2020) | - |

WHO - World Health Organization, OECD - Organisation for Economic Co-operation and Development, UNESCO - United Nations Educational, Scientific and Cultural Organization, UNICEF - United Nations Children’s Fund.

Australian Institute of Health and Welfare. (2020). *Australia’s children*. Cat. no. CWS 69. Canberra: AIHW. https://doi.org/10.25816/5ebca4d0fa7dd

Bradshaw, J., Hoelscher, P., & Richardson, D. (2007). An index of Child Well-Being in the European Union. *Social Indicators Research*, *80*(1), 133–177. https://doi.org/10.1007/s11205-006-9024-z

Chang, Y. J., Lehmann, A., Winter, L., & Finkbeiner, M. (2018). The Sustainable Child Development Index (SCDI) for countries. *Sustainability (Switzerland)*, *10*(5). https://doi.org/10.3390/su10051563

Clark, H., Coll-Seck, A. M., Banerjee, A., Peterson, S., Dalglish, S. L., Ameratunga, S., … Costello, A. (2020). A future for the world’s children? A WHO–UNICEF–Lancet Commission. *The Lancet*, *395*(10224), 605–658. https://doi.org/10.1016/S0140-6736(19)32540-1

El-Kogali, S., & Krafft, C. (2015). *Expanding Opportunities for the Next Generation: Early Childhood Development in the Middle East and North Africa*. Directions in Development. Washington, DC: World Bank. License: Creative Commons Attribution CC BY 3.0 IGO. https://doi.org/10.1596/978-1-4648-0323-9

Ford, N. D., & Stein, A. D. (2016). Risk factors affecting child cognitive development: A summary of nutrition, environment, and maternal-child interaction indicators for sub-Saharan Africa. *Journal of Developmental Origins of Health and Disease*, *7*(2), 197–217. https://doi.org/10.1017/S2040174415001427

Keating, K., Cole, P., & Schaffner, M. (2020). *The State of Babies Yearbook: 2020*. Washington, DC: ZERO TO THREE. Retrieved from https://stateofbabies.org/wp-content/uploads/2020/06/State-of-Babies-2020-Full-Yearbook-061820.pdf

Köhler, L., & Eriksson, B. (2018). A Child Health Index for Sweden’s 290 Municipalities. *Child Indicators Research*, *11*(6), 1889–1906. https://doi.org/10.1007/s12187-017-9515-2

Maria Cecília Souto Vidigal Foundation. (2020). Early Childhood First in the Municipality (Primeira Infância Primeiro no Município). Retrieved from https://primeirainfanciaprimeiro.fmcsv.org.br/

National Early Childhood Network, & Andi Communication and Rights. (2020). *Observatory of the Early Childhood Legal Framework (Observatório do Marco Legal da Primeira Infância)*. Retrieved from https://rnpiobserva.org.br/

Nossa São Paulo Network, & Bernard van Leer Foundation. (2020). Early childhood inequality map 2020 (Mapa da desigualdade da primeira infância 2020). Retrieved from https://www.nossasaopaulo.org.br/2020/02/12/mapa-da-desigualdade-da-primeira-infancia-2020-e-lancado/

OECD. (2009). *Doing better for children*. OECD Publishing, Paris. https://doi.org/10.1787/9789264059344-en

OECD. (2015). *How’s Life? 2015: Measuring Well-being*. OECD Publishing, Paris. https://doi.org/10.1787/how_life-2015-en

Save the Children. (2009). *State of the world’s mothers 2009: Investing in the Early Years*. Retrieved from https://www.savethechildren.org/content/dam/usa/reports/advocacy/sowm/sowm-2009.pdf

Save the Children. (2012). Nutrition in the First 1,000 Days. In *State of the World’s Mothers 2012*. Retrieved from https://www.savethechildren.org/content/dam/usa/reports/advocacy/sowm/sowm-2012.pdf

Save the Children. (2016). *Every Last Child:The children the world chooses to forget*. Retrieved from https://www.savethechildren.org/content/dam/usa/reports/advocacy/EVERY-LAST-CHILD.PDF

Save the Children. (2018). *U.S. Complement to the End of Childhood Report 2017: Growing up in America*. Retrieved from https://resourcecentre.savethechildren.net/node/15259/pdf/2018-end-of-childhood-report-us.pdf

Save the Children. (2019). Changing Lives in Our Lifetime. *Global Childhood Report 2019*, 68. Retrieved from https://www.savethechildren.org/content/dam/usa/reports/advocacy/global-childhood-report-2019-pdf.pdf

State System of Data Analysis Foundation, & São Paulo State Government Planning and Management Department. (2015). São Paulo Early Childhood Index (Índice Paulista da Primeira Infância). Retrieved from http://www.ippih.seade.gov.br/frontend/#/

The World Bank. (2013). What Matters Most for Early Childhood Development: A Framework Paper. *Systems Approach for Better Education Results (SABER) Working Paper Series*, 4–59. Retrieved from http://wbgfiles.worldbank.org/documents/hdn/ed/saber/supporting_doc/Background/ECD/Framework_SABER-ECD.pdf

UNESCO. (2014). *Holistic Early Childhood Development Index (HECDI) Framework: A technical guide*. Retrieved from https://unesdoc.unesco.org/ark:/48223/pf0000229188

UNICEF. (2001). *O Índice de Desenvolvimento Infantil (IDI)*. (Idi), 58–69. Retrieved from https://cursosextensao.usp.br/pluginfile.php/243758/mod_resource/content/0/IDI.pdf

United Nations Children’s Fund, & Countdown to 2030. (2020). *Country profiles for early childhood development*. Retrieved from https://nurturing-care.org/resources/country-profiles/

United Nations Children’s Fund, & World Health Organization. (2017). *Tracking Progress towards Universal Coverage for Women’s, Children’s and Adolescents’ Health. The 2017 Report*. Washington, DC. Licence: CC BY-NC-SA 3.0 IGO. Retrieved from http://countdown2030.org

University of Brasilia, Federal University of Bahia, & Yale School of Public Health. (2020). *Early Childhood Friendly Municipal Index (Índice Município Amigo da Primeira Infância - IMAPI)*. Retrieved from https://imapi.org/

Urke, H. B., Mittelmark, M. B., Amugsi, D. A., & Matanda, D. J. (2018). Resources for nurturing childcare practices in urban and rural settings: Findings from the Colombia 2010 Demographic and Health Survey. *Child: Care, Health and Development*, *44*(4), 572–582. https://doi.org/10.1111/cch.12570

van den Heuvel, M., Hopkins, J., Biscaro, A., Srikanthan, C., Feller, A., Bremberg, S., … Williams, R. (2013). A comparative analysis of early child health and development services and outcomes in countries with different redistributive policies. *BMC Public Health*, *13*(1). https://doi.org/10.1186/1471-2458-13-1049

World Health Organization. (2010). *Child and adolescent health and development: progress report 2009: highlights*. Retrieved from http://whqlibdoc.who.int/publications/2010/9789241599368_eng.pdf

World Health Organization, United Nations Children’s Fund, & World Bank Group. (2018). *Nurturing care for early child development: a framework for helping children survive and thrive to transform health and human potential.* Geneva: World Health Organization; 2018. Licence: CC BY-NC-SA 3.0 IGO.

Wu, K. B., Young, M. E., & Cai, J. (2012). *Early child development in China: Breaking the Cycle of Poverty and Improving Future Competitiveness*. Washington DC: World Bank. License: Creative Commons Attribution CC BY 3.0. https://doi.org/10.1596/978-0-8213-9564-6
