# Supplementary material for: Population‐based approaches for monitoring the nurturing care environment for early childhood development: A scoping review
Source: Matern Child Nutr. 2021 Nov 4;18(Suppl 2):e13276. doi: 10.1111/mcn.13276 (PMC8968941; doi:10.1111/mcn.13276)
Supplement: Supplementary file 3 — Data S2. Supporting Information [file MCN-18-e13276-s001.docx]

**Supporting Information.** Search Strategy.

**Databases**

**Pubmed:**

((((("child development"[All Fields] OR (("child development"[MeSH Terms] OR "child development"[All Fields])) OR "childhood development"[All Fields]) OR "infant development"[All Fields]) AND (("environment*"[All Fields] OR ((((("environ"[All Fields] OR "environment"[MeSH Terms]) OR "environment"[All Fields]) OR "environments"[All Fields]) OR "environment s"[All Fields]) OR "environs"[All Fields])) OR "nurturing care"[All Fields])) AND (((((((((((("tool*"[All Fields] OR "index*"[All Fields]) OR "indicator*"[All Fields]) OR "model*"[All Fields]) OR "measurement method*"[All Fields]) OR "environmental indicator*"[All Fields]) OR (((("environmental indicators"[MeSH Terms] OR "environmental indicators"[All Fields]) OR "environmental indicator"[All Fields])) OR "instrument*"[All Fields]) OR "monitoring"[All Fields]) OR "monitoring system*"[All Fields]) OR "social indicator*"[All Fields]) OR "public health surveillance"[All Fields]) OR (("public health surveillance"[MeSH Terms] OR "public health surveillance"[All Fields]))) AND ((((((((("developing countr*"[All Fields] OR (("developing countries"[MeSH Terms] OR "developing countries"[All Fields])) OR "developed countr*"[All Fields]) OR (("developed countries"[MeSH Terms] OR "developed countries"[All Fields])) OR "countr*"[All Fields]) OR "state*"[All Fields]) OR "municipalit*"[All Fields]) OR (("cities"[MeSH Terms] OR "cities"[All Fields]) OR "city"[All Fields])) OR (("cities"[MeSH Terms] OR "cities"[All Fields]) OR "city s"[All Fields])) OR "town*"[All Fields])

**Virtual Health Library (VHL)**

tw:((tw:("child development" OR "childhood development" OR "infant development")) AND (tw:("environment*" OR "nurturing care")) AND (tw:(tool* OR index* OR indicator* OR model* OR "measurement method" OR "environmental indicators" OR instrument* OR "monitoring" OR "monitoring system" OR "social indicators" OR “public health surveillance”)) AND (tw:(“developing countries” OR "developed countries" OR countr* OR state* OR municipalit* OR city OR cities OR town*)))

**Websites**

**Inter-American Development Bank (IDB)**

"child development"

**Grand Challenges Bill & Melinda Gates:**

child development

**Grand Challenges Canada:**

child development

**United States Agency for International Development (USAID)**

("child development" OR "childhood development" OR "infant development") AND (tool OR index OR indicator)

**United Nations Children's Fund (UNICEF) new website (**[**https://www.unicef.org/**](https://www.unicef.org/)**)**

"childhood development"

**United Nations Children's Fund (UNICEF) old website**

**(**[**https://www.unicef.org/search/search.php?q=child%20development**](https://www.unicef.org/search/search.php?q=child%20development)**)**

"child development index" OR "child development tool" OR "child development indicator" OR "childhood development index" OR "childhood development tool" OR "childhood development indicator" OR "infant development index" OR "infant development tool" OR "infant development indicator"

**World Bank (**[**https://www.worldbank.org/**](https://www.worldbank.org/)**)**

(child development index OR child development tool OR child development indicator OR childhood development index OR childhood development tool OR childhood development indicator OR infant development index OR infant development tool OR infant development indicator)

**World Bank eLibrary**

"child development" OR "childhood development" OR "infant development"

AND

"environment" OR environments OR environmental OR "nurturing care"

AND

tool OR tools OR index OR indexes OR indicator OR indicators OR model OR models OR "measurement method" OR "measurement methods" OR "environmental indicator" OR "environmental indicators" OR instrument OR instruments OR "monitoring" OR "monitoring system" OR "monitoring systems" OR “social indicator” OR “social indicators” OR “public health surveillance”

AND

“developing country” “developing countries” OR “developed country” OR “developed countries” OR country OR countries OR state OR states OR municipality OR municipalities OR city OR cities OR town OR towns

**World Health Organization (WHO)**

("child development" OR "childhood development" OR "infant development") AND ("environment" OR environments OR environmental OR "nurturing care") AND (tool OR tools OR index OR indexes OR indicator OR indicators OR model OR models OR "measurement method" OR "measurement methods" OR "environmental indicator" OR "environmental indicators" OR instrument OR instruments OR "monitoring" OR "monitoring system" OR "monitoring systems" OR “social indicator” OR “social indicators” OR “public health surveillance”) AND (“developing country” “developing countries” OR “developed country” OR “developed countries” OR country OR countries OR state OR states OR municipality OR municipalities OR city OR cities OR town OR towns)

**Organization for Economic Co-operation and Development (OECD) iLibrary**

"child development index" OR "child development tool" OR "child development indicator" OR "childhood development index" OR "childhood development tool" OR "childhood development indicator" OR "infant development index" OR "infant development tool" OR "infant development indicator"

**Pan American Health Organization (PAHO)**

child development

**Save the Children**

("child development" OR "childhood development" OR "infant development") AND ("environment" OR environments OR environmental OR "nurturing care") AND (tool OR tools OR index OR indexes OR indicator OR indicators OR model OR models OR "measurement method" OR "measurement methods" OR "environmental indicator" OR "environmental indicators" OR instrument OR instruments OR "monitoring" OR "monitoring system" OR "monitoring systems" OR “social indicator” OR “social indicators” OR “public health surveillance”) AND (“developing country” “developing countries” OR “developed country” OR “developed countries” OR country OR countries OR state OR states OR municipality OR municipalities OR city OR cities OR town OR towns)
