# Supplementary material for: Population‐based approaches for monitoring the nurturing care environment for early childhood development: A scoping review
Source: Matern Child Nutr. 2021 Nov 4;18(Suppl 2):e13276. doi: 10.1111/mcn.13276 (PMC8968941; doi:10.1111/mcn.13276)
Supplement: Supplementary file 4 — Data S3. Supporting Information [file MCN-18-e13276-s003.docx]

**Supporting Information.** Evaluation of the groups of indicators according to the Sustainable Development Goals covered.

| **Domain** | **Group (n = indicators)** | **Sustainable Development Goals (SDGs)** | | | | | | | | | | | | | | | | |
| --- | --- | --- | --- | --- | --- | --- | --- | --- | --- | --- | --- | --- | --- | --- | --- | --- | --- | --- |
|  |  | **01** | **02** | **03** | **04** | **05** | **06** | **07** | **08** | **09** | **10** | **11** | **12** | **13** | **14** | **15** | **16** | **17** |
| **Good Health** | Child mortality (n = 41) * |  |  | ● |  |  |  |  |  |  |  |  |  |  |  |  |  |  |
|  | Suicide mortality (n = 3) |  |  | ● |  |  |  |  |  |  |  |  |  |  |  |  |  |  |
|  | Birth weight (n = 19) * |  |  | ● |  |  |  |  |  |  |  |  |  |  |  |  |  |  |
|  | Prenatal care (n = 14) |  |  | ● |  |  |  |  |  |  |  |  |  |  |  |  |  |  |
|  | Delivery Conditions (n = 14) |  |  | ● |  |  |  |  |  |  |  |  |  |  |  |  |  |  |
|  | Adolescent pregnancy (n = 17) |  |  | ● |  | ● |  |  |  |  |  |  |  |  |  |  |  |  |
|  | HIV (n = 6) |  |  | ● |  |  |  |  |  |  |  |  |  |  |  |  |  |  |
|  | Child vaccination / immunization (n = 29) * |  |  | ● |  |  |  |  |  |  |  |  |  |  |  |  |  |  |
|  | Diarrhoea treatment (n = 9) |  |  | ● |  |  |  |  |  |  |  |  |  |  |  |  |  |  |
|  | Respiratory diseases (n = 11) |  |  | ● |  |  |  |  |  |  |  |  |  |  |  |  |  |  |
|  | Preterm (n = 3) |  |  | ● |  |  |  |  |  |  |  |  |  |  |  |  |  |  |
|  | Congenital syphilis (n = 3) |  |  | ● |  |  |  |  |  |  |  |  |  |  |  |  |  |  |
|  | Postnatal Care (n = 5) |  |  | ● |  |  |  |  |  |  |  |  |  |  |  |  |  |  |
|  | Coverage of health services (n = 7) |  |  | ● |  |  |  |  |  |  |  |  |  |  |  |  |  |  |
|  | Other diseases or conditions (n = 14) |  |  | ● |  |  |  |  |  |  |  |  |  |  |  |  |  |  |
|  | Adolescent mortality (n = 3) |  |  | ● |  |  |  |  |  |  |  |  |  |  |  |  |  |  |
|  | Malaria infection (n = 5) |  |  | ● |  |  |  |  |  |  |  |  |  |  |  |  |  |  |
|  | Health behavior (n = 6) |  |  | ● |  |  |  |  |  |  |  |  |  |  |  |  |  |  |
|  | Maternal mortality (n = 12) |  |  | ● |  |  |  |  |  |  |  |  |  |  |  |  |  |  |
|  | Child and young people well-being (n = 9) |  |  | ● |  |  |  |  |  |  |  |  |  |  |  |  |  |  |
|  | Policies, laws, programs and action for newborn, child and maternal health (n = 14) |  |  | ● |  | ● |  |  |  |  |  |  |  |  |  |  | ● | ● |
|  | Family planning (n = 4) |  |  |  |  | ● |  |  |  |  |  |  |  |  |  |  |  |  |
|  | Access and quality of health facilities (n = 17) |  |  | ● |  |  |  |  |  |  | ● |  |  |  |  |  |  |  |
|  | Expenditure on health (n = 8) |  |  | ● |  |  |  |  |  |  |  |  |  |  |  |  |  | ● |
| **Adequate Nutrition** | Child Nutritional Status (n = 29) * |  | ● |  |  |  |  |  |  |  |  |  |  |  |  |  |  |  |
|  | Women Nutritional Status (n = 5) |  | ● |  |  |  |  |  |  |  |  |  |  |  |  |  |  |  |
|  | Breastfeeding (n = 19) |  | ● |  |  |  |  |  |  |  |  |  |  |  |  |  |  |  |
|  | Diet characteristics (n = 10) |  | ● |  |  |  |  |  |  |  |  |  |  |  |  |  |  |  |
|  | Anemia (n = 4) |  | ● |  |  |  |  |  |  |  |  |  |  |  |  |  |  |  |
|  | Food Security (n = 3) |  | ● |  |  |  |  |  |  |  |  |  |  |  |  |  |  |  |
|  | Micronutrient supplementation (n = 4) |  | ● |  |  |  |  |  |  |  |  |  |  |  |  |  |  |  |
|  | Food fortification (n = 4) |  | ● |  |  |  |  |  |  |  |  |  |  |  |  |  |  |  |
|  | Lifetime cost of growth deficit in early childhood (n = 1) |  | ● | ● |  |  |  |  | ● |  | ● |  |  |  |  |  |  |  |
|  | Policies, laws and programs that support adequate nutrition (n = 8) | ● | ● | ● |  |  |  |  |  |  |  |  | ● |  |  |  | ● | ● |
| **Responsive Caregiving** | Development milestones (n = 19) |  |  | ● | ● |  |  |  |  |  |  |  |  |  |  |  |  |  |
|  | Inadequate supervision (n = 6) |  |  | ● | ● |  |  |  |  |  |  |  |  |  |  |  |  |  |
|  | Caregivers well-being (n = 8) |  |  | ● |  |  |  |  |  |  |  |  |  |  |  |  |  |  |
|  | Home visits programs (n = 6) |  |  | ● |  |  |  |  |  |  |  |  |  |  |  |  |  |  |
|  | Programs, services and strategy for ECD (n = 16) |  |  | ● | ● |  |  |  |  |  |  |  |  |  |  |  |  |  |
|  | Family resilence (n = 1) | ● |  |  |  |  |  |  |  |  | ● | ● |  |  |  |  | ● |  |
|  | Social Support (n = 2) |  |  |  |  |  |  |  |  |  |  | ● |  |  |  |  | ● |  |
|  | Child development screening (n = 3) |  |  | ● | ● |  |  |  |  |  |  |  |  |  |  |  |  |  |
|  | Recognizing and responding to illness and danger signs (n = 1) |  |  | ● |  |  |  |  |  |  |  |  |  |  |  |  |  |  |
| **Opportunities for early learning** | Caregiver-child interactions (n = 14) |  |  |  | ● |  |  |  |  |  |  |  |  |  |  |  |  |  |
|  | Access to educational supplies (n = 8) |  |  |  | ● |  |  |  |  |  |  |  |  |  |  |  |  |  |
|  | Access to education and enrollment (n = 50) |  |  |  | ● |  |  |  |  |  | ● |  |  |  |  |  |  |  |
|  | Educational deprivation (n = 2) |  |  |  | ● |  |  |  |  |  |  |  |  |  |  |  |  |  |
|  | School characteristics (n = 5) |  |  |  | ● |  |  |  |  |  |  |  |  |  |  |  |  |  |
|  | School attendance (n = 9) |  |  |  | ● |  |  |  |  |  | ● |  |  |  |  |  |  |  |
|  | Training or qualification of child care staff (n = 12) |  |  |  | ● |  |  |  |  |  |  |  |  |  |  |  |  |  |
|  | Child care staff proportion (n = 8) |  |  |  | ● |  |  |  |  |  |  |  |  |  |  |  |  |  |
|  | Schools environment (n = 6) |  |  |  | ● |  |  |  |  |  |  | ● |  |  |  |  |  |  |
|  | Child educational perfomance (n = 21) |  |  |  | ● |  |  |  |  |  | ● |  |  |  |  |  |  |  |
|  | Child feelings about school (n = 6) |  |  |  | ● |  |  |  |  |  |  |  |  |  |  |  | ● |  |
|  | Out-of-school children (n = 6) |  |  |  | ● | ● |  |  |  |  | ● |  |  |  |  |  |  |  |
|  | Support to education (n = 15) |  |  |  | ● |  |  |  |  |  |  |  |  |  |  |  | ● | ● |
| **Security and safety** | Violence (n = 23) * |  |  |  |  | ● |  |  |  |  |  |  |  |  |  |  | ● |  |
|  | Birth registration (n = 8) |  |  |  |  |  |  |  |  |  | ● |  |  |  |  |  | ● |  |
|  | Sanitation (n = 18) |  |  |  |  |  | ● |  |  |  |  |  |  |  |  |  |  |  |
|  | Cash transfer programs (n = 5) | ● | ● |  |  | ● |  |  |  |  | ● |  |  |  |  |  | ● |  |
|  | Environment (n = 4) |  |  |  |  |  | ● | ● |  |  |  | ● | ● | ● |  |  |  |  |
|  | Alchool and smoking (n = 12) |  |  | ● |  |  |  |  |  |  |  |  |  |  |  |  |  |  |
|  | Drugs / Substance use (n = 2) |  |  | ● |  |  |  |  |  |  |  |  |  |  |  |  |  |  |
|  | Insecticide treated nets and spraying (n = 5) |  |  | ● |  |  |  |  |  |  |  | ● |  |  |  |  |  |  |
|  | Bullying (n = 4) |  |  |  |  |  |  |  |  |  |  |  |  |  |  |  | ● |  |
|  | Child labor (n = 2) | ● |  |  |  | ● |  |  | ● |  |  |  |  |  |  |  | ● |  |
|  | Home environment (n = 13) | ● |  |  |  |  |  |  |  |  |  | ● |  |  |  |  |  |  |
|  | Neighborhood environment (n = 9) |  |  |  |  |  | ● | ● |  |  | ● | ● | ● |  |  |  | ● | ● |
|  | Children in foster care (n = 6) | ● |  |  |  | ● |  |  |  |  |  |  |  |  |  |  | ● |  |
|  | Child or adolescent marriage (n = 3) |  |  |  |  | ● |  |  |  |  |  |  |  |  |  |  |  |  |
|  | Homelessness (n = 1) | ● |  |  |  |  |  |  |  |  | ● | ● |  |  |  |  |  |  |
|  | Traffic accidents (n = 2) |  |  | ● |  |  |  |  |  |  |  | ● |  |  |  |  |  |  |
|  | Access to social assistance services (n = 1) | ● |  | ● |  |  |  |  |  |  |  |  |  |  |  |  | ● |  |
|  | Electricity (n = 1) |  |  |  |  |  |  | ● |  |  |  |  |  |  |  |  |  |  |
|  | Child and adolescent sexual behaviour (n = 2) |  |  | ● |  | ● |  |  |  |  |  |  |  |  |  |  |  |  |
|  | Hygiene habits (n = 1) |  |  | ● |  |  | ● |  |  |  |  |  |  |  |  |  |  |  |
|  | Insurance coverage (n = 1) |  |  | ● |  |  |  |  |  |  | ● |  |  |  |  |  | ● | ● |
|  | Injuries or mortality due to environmental factors (n = 3) |  |  | ● |  |  |  |  |  |  |  |  |  |  |  |  | ● |  |
|  | Vulnerability (n = 27) * | ● | ● |  |  | ● |  |  | ● |  | ● |  |  |  |  |  |  |  |
|  | Health Insurance (n = 7) |  |  | ● |  |  |  |  |  |  |  |  |  |  |  |  |  |  |
|  | Discipline (n = 1) |  |  |  |  |  |  |  |  |  |  |  |  |  |  |  | ● |  |
|  | Family structure (n = 5) |  |  |  |  |  |  |  |  |  |  |  |  |  |  |  |  |  |
|  | Imigrants and refugees (n = 3) |  |  |  |  |  |  |  |  |  | ● |  |  |  |  |  |  |  |
|  | Adults education (n = 8) | ● |  |  | ● | ● |  |  | ● |  | ● |  |  |  |  |  |  |  |
|  | Adult unemployment (n = 4) | ● |  |  |  |  |  |  | ● |  |  |  |  |  |  |  |  |  |
|  | Adverse Childhood Experiences (ACEs) (n = 2) | ● | ● | ● | ● | ● | ● | ● |  |  | ● | ● |  |  |  |  | ● | ● |
|  | Parental leave (n = 6) |  |  |  |  | ● |  |  |  |  | ● |  |  |  |  |  | ● |  |
|  | Policies, laws, and programs for social protection (n = 18) | ● |  |  |  |  |  |  |  |  | ● |  |  |  |  |  | ● |  |
|  | Policy governance (n = 3) |  |  |  |  |  |  |  |  |  |  |  |  |  |  |  | ● | ● |
| **Demographic characteristics** | Fertility rate (n = 2) |  |  |  |  |  |  |  |  |  |  |  |  |  |  |  |  |  |
|  | Births (n = 4) |  |  |  |  |  |  |  |  |  |  |  |  |  |  |  |  |  |
|  | Child and Adolescent Population (n = 21) |  |  |  |  |  |  |  |  |  |  |  |  |  |  |  |  |  |
|  | Total Population (n = 9) |  |  |  |  |  |  |  |  |  |  |  |  |  |  |  |  |  |
|  | Geographic scope (n = 6) |  |  |  |  |  |  |  |  |  |  |  |  |  |  |  |  |  |
|  | Age and Sex of child or mother (n = 5) |  |  |  |  |  |  |  |  |  |  |  |  |  |  |  |  |  |
|  | Race/ethnicity (n = 1) |  |  |  |  |  |  |  |  |  |  |  |  |  |  |  |  |  |
|  | Socioeconomic indicators (indexes) (n = 17) | ● | ● |  |  | ● |  |  | ● |  | ● |  |  |  |  |  |  |  |
|  | Maternal occupation (n = 2) |  |  |  |  | ● |  |  | ● |  |  |  |  |  |  |  |  |  |
|  | Politics and civic participation (n = 5) |  |  |  |  |  |  |  |  |  | ● |  |  |  |  |  | ● |  |
|  | Life expectancy (n = 1) | ● |  | ● |  |  |  |  |  |  | ● |  |  |  |  |  |  |  |

1: No Poverty; 2: Zero Hunger; 3. Good Health and Well-Being; 4. Quality Education; 5. Gender Equality; 6. Clean Water and Sanitation; 7. Affordable and Clean Energy; 8. Decent Work and Economic Growth; 9. Industry, Innovation and Infrastructure; 10. Reduced Inequalities; 11. Sustainable Cities and Communities; 12. Responsible Consumption and Production; 13. Climate Action; 14. Life Below Water; 15. Life on Land; 16. Peace, Justice and Strong Institutions; 17. Partnerships for The Goals.
